# Size Matters: The Influence of Patient Size on Antibiotics Exposure Profiles in Critically Ill Patients on Continuous Renal Replacement Therapy

**DOI:** 10.3390/antibiotics10111390

**Published:** 2021-11-12

**Authors:** Soo-Min Jang, Alex R. Shaw, Bruce A. Mueller

**Affiliations:** 1 Department of Pharmacy Practice, Loma Linda University School of Pharmacy, Loma Linda, CA 92350, USA; 2 Medical Strategist, Ironwood Pharmaceuticals, Boston, MA 02110, USA; arshaw89@gmail.com; 3 Department of Clinical Pharmacy, University of Michigan College of Pharmacy, Ann Arbor, MI 48109, USA; muellerb@med.umich.edu

**Keywords:** renal replacement therapy, Monte Carlo simulation, antibiotics, pharmacokinetics, pharmacodynamics

## Abstract

(1) Purpose of this study: To determine whether patient weight influences the probability of target attainment (PTA) over 72 h of initial therapy with beta-lactam (cefepime, ceftazidime, piperacillin/tazobactam) and carbapenem (imipenem, ertapenem, meropenem) antibiotics in the critical care setting. This is the first paper to address the question of whether patient size affects antibiotic PTA in the ICU. (2) Methods: We performed a post hoc analysis of Monte Carlo simulations conducted in virtual critically ill patients receiving antibiotics and continuous renal replacement therapy. The PTA was calculated for each antibiotic on the following pharmacodynamic (PD) targets: (a) were above the target organism’s minimum inhibitory concentration (≥%fT≥1×MIC), (b) were above four times the MIC (≥%fT≥4×MIC), and (c) were always above the MIC (≥100%fT≥MIC) for the first 72 h of antibiotic therapy. The PTA was analyzed in patient weight quartiles [Q1 (lightest)-Q4 (heaviest)]. Optimal doses were defined as the lowest dose achieving ≥90% PTA. (3) Results: The PTA for fT≥1×MIC led to similarly high rates regardless of weight quartiles. Yet, patient weight influenced the PTA for higher PD targets (100%fT≥MIC and fT≥4×MIC) with commonly used beta-lactams and carbapenems. Reaching the optimal PTA was more difficult with a PD target of 100%fT≥MIC compared to fT≥4×MIC. (4) Conclusions: The Monte Carlo simulations showed patients in lower weight quartiles tended to achieve higher antibiotic pharmacodynamic target attainment compared to heavier patients.

## 1. Introduction

Continuous renal replacement therapy (CRRT) is the preferred renal replacement therapy (RRT) over intermittent hemodialysis in patients with acute kidney injury (AKI) due to hemodynamic instability [1]. The multicenter study Veterans Affairs/National Institutes of Health Acute Renal Failure Trial Network Study (ATN trial) showed that there was no difference in clinical outcomes when patients received less-intensive or intensive effluent rates for CRRT [2]. Since the antibiotic doses were used in both intensity arms, some suggested that patients with intensive CRRT may have had lower overall antibiotic exposures due to a higher drug removal rate [3,4]. Our previous study showed there were no significant differences in the probability of target attainment (PTA) between less-intensive (20–25 mL/kg/h) vs. intensive (35–45 mL/kg/h) effluent rate arms [5].

The combination of AKI and aggressive fluid resuscitation for sepsis leads to a considerable amount of fluid weight gain, increasing the volume of distribution (Vd) in drugs [6]. Increased Vd leads to a lower plasma concentration, requiring higher doses of a drug. This is noteworthy because altered Vd in AKI patients receiving CRRT can cause high interindividual and interoccasion variability in antibiotic serum concentrations [7]. For example, interindividual variability was noted with piperacillin and tazobactam trough levels by ≥123-fold and ≥192-fold, respectively, in critically ill patients [8]. Moreover, the majority of clinical studies that derived dosing recommendations do not include larger patients (>100 kg), and obesity is a well-known risk factor of antibiotic therapy failure [9,10].

Antimicrobial activity is impacted by multiple factors, including drug dose regimen, potency of the drug against a specific organism, and pharmacokinetic (PK) parameters. For beta-lactam agents, in vitro and clinical studies suggest that maintaining free serum concentrations at least four times as high as the organism’s minimum inhibitory concentration (MIC) (fT≥4×MIC) optimizes bactericidal activity and clinical response in critically ill patients compared to less stringent pharmacodynamic targets [11,12,13]. Moreover, clinical outcomes were superior when the PD target maintained free drug concentrations above the 1×MIC (fT≥1×MIC) level for 100% of the dosing interval in critically ill patients [14]. The objective of this post-hoc study was to determine PTA over the first 72 h of commonly prescribed doses of beta-lactams (cefepime, ceftazidime, and piperacillin/tazobactam) and carbapenems (imipenem, meropenem) in different patient weight quartiles using Monte Carlo simulation (MCS) techniques.

## 2. Results

The PTA rates in overall (for all 10,000 virtual patients) and in different weight quartiles [Q1 (lightest) to Q4 (heaviest)] for cefepime, ceftazidime, piperacillin, and tazobactam are reported in Table 1. Table 2 lists the PTA for overall virtual patients and different weight quartiles for the meropenem, imipenem, and ertapenem dosing regimens. Three different pharmacodynamic targets were assessed, from the least stringent %fT≥1×MIC to the most stringent target of 100%fT≥MIC.

As reported in Table 1 (cefepime, ceftazidime, piperacillin/tazobactam), the PTA against *P. aeruginosa* consistently decreases as the weight quartile increases. The PTA in less-intensive CRRT effluent rate arms was higher than the PTA in intensive CRRT effluent rate arms for all drugs. Nevertheless, these differences were usually small within any weight quartile for any drug. Table 2 illustrates similar findings for carbapenem antibiotics. With a few exceptions, the carbapenem PTA decreased as the weight quartiles increased. The intensity of the CRRT effluent rate also influenced the PTA such that lower PTA rates were observed in the intensive CRRT than in the analogous lower CRRT intensity groups. Again, the differences observed with CRRT intensity were not large. Two drug dosing regimens (imipenem 1 g every 8 h and meropenem 2 g every 12 h) showed a different trend, namely that their PTAs increased as the weight quartile increased.

## 3. Discussion

This is the first MCS to examine the influence of subject weights on antibiotic PTA in patients receiving CRRT. Our hypothesis for the present study was that antibiotic exposures will be significantly lower (resulting in a lower PTA) in heavier virtual critically ill patients (obesity and/or fluid overloaded) receiving CRRT when the same daily antibiotic dose is used. Our results showed virtual patients who were in Q1 (the lightest quartile) had a higher PTA for its PD target; the PTA gradually decreased as the weight quartile increased [the heaviest (Q4) had the lowest PTA] for all drugs in this study (cefepime, ceftazidime, piperacillin, tazobactam, ertapenem, imipenem, and meropenem) with few exceptions.

The lowest modeled cefepime dosing regimen (1 g every 12 h) met acceptable PTA rates at the least stringent (%fT≥1×MIC) target but poor PTA achievement in both the less-intensive and intensive CRRT groups for the more stringent PD targets (fT≥4×MIC and 100%fT≥MIC). For cefepime, the PTA significantly decreased as the weight quartiles (heavier patients) increased. For example, the overall PTA for 100%fT≥MIC with cefepime 2 g every 12 h in the less-intensive group was 56.3%. Yet, in the first quartile (weight: 40–70 kg) and the last quartile (weight: 95–180 kg) in the less-intensive group achieved PTA values of 90.3% and 17.3%, respectively. Ceftazidime followed a similarly lower PTA with a higher weight trend. Ceftazidime 2 g every 12 h, in the less-intensive group for the PD target of 100%fT≥1×MIC, yielded an overall PTA of 81.1%. However, it exhibited large differences between weight quartiles: 93.2% (in Q1: 40–70 kg) and 63.5% (in Q4: 95–187 kg). Weights influenced piperacillin/tazobactam, as the PTA decreased as the weight quartile increased. For instance, the overall PTA was 60%, but Q1 (40–71 kg) and Q4 (95–184 kg) were 77.9% and 39.8%, respectively, for the PD target of 100%fT≥MIC with piperacillin 4 g every 6 h.

In our study, a few carbapenem dosing regimens demonstrated interesting results, for example, ertapenem 1 g every 24 h with the PD target of 100%fT≥MIC. Subjects in the intensive CRRT arm in Q1 (the lightest) exhibited the lowest PTA compared to larger virtual patients. The PTAs were: Q1 82%, Q2 91.8%, Q3 93.4%, and Q4 83.7%. One potential explanation is that Q1 subjects had the smallest Vd, which may have led to a higher relative drug clearance by intensive CRRT. Imipenem also showed unusual results within the 1 g every 8 h dosing regimen model. The PTA increased as the weight quartiles increased for the PD target of 100%fT≥MIC: PTA Q1 66%; Q2 81%; Q3 89%, and Q4 94% in the intensive CRRT group. With further PK analysis with this cohort, the mean Vd for Q1 was 0.33 L/kg (20.39 L) and in Q4 was 0.37 L/kg (40.36 L). Moreover, the mean nonrenal clearance (CL_NR_) for Q1 subjects was 98.5 mL/min when CL_NR_ for Q4 subjects was 97.9 mL/min. This phenomenon (increased PTA with higher weight) may be explained by a combination of smaller Vd leading to more drug removal by CRRT and higher CL_NR_ in the Q1 cohort. The other standard dosing regimens for carbapenem results were consistent with our hypothesis (lower PTA with higher weight quartiles).

This study is consistent with Hites et al. [15], who evaluated beta-lactam standard dosing regimens in critically ill patients (both obese and nonobese patients). They found the standard dosing regimens resulted in subtherapeutic plasma concentrations in 32% of their patients and supratherapeutic plasma concentrations in 25% overall. It was evident for meropenem that more obese patients had subtherapeutic antibiotic concentrations compared to nonobese patients (35% vs. 0%, *p* = 0.02) [15]. The authors did not find statistical differences between obese and nonobese patients for cefepime and piperacillin/tazobactam. Lastly, patients receiving CRRT were more likely to result in supratherapeutic levels than patients who were not receiving CRRT (44.1% vs. 8.8%; *p* = 0.002) in this study. Moreover, obese patients receiving CRRT were more likely to have supratherapeutic levels compared to nonobese patients receiving CRRT.

Taccone et al. [16] shared a case report that illustrated that obese patients require a much higher antibiotic dosing regimen compared to nonobese patients. This case report was regarding a patient who had a body mass index (BMI) of 35 who presented with septic shock due to extensively drug-resistant *P. aeruginosa*. The PD target was 40%T≥4×MIC, and the standard meropenem dosing regimen did not reach the PD target. The patient required meropenem of 12 g/d (3 g every 6 h with 3 h extended infusion), which resulted in meropenem resolution without any adverse events and no abnormal electroencephalogram.

Cheatham et al. [17] evaluated pharmacokinetics and pharmacodynamics with meropenem use in morbidly obese patients. Nine patients were included with a total body weight of 152.3 ± 31.0 kg (ideal body weight: 60.3 ± 10.6 kg) and a BMI of 54.7 ± 8.6 kg/m^2^. The authors found appropriate meropenem dosing regimens for morbidly obese patients were 1 g every 8 h, 2 g every 8 h, 500 mg every 6 h, and 1 g every 6 h when the PD target was 40%fT≥1×MIC (2 mg/mL). For a more stringent PD target (40%fT≥4×MIC), 2 g every 8 h and 1 g every 6 h were necessary for this special population. Even though this study did not include critically ill patients receiving CRRT, it highlights that morbidly obese patients require a higher meropenem dose.

This study has several limitations, including not having BMI information since the study was based on MCS (virtual patients). The PK parameters were derived in different patient populations other than the American patients (ATN trial). However, our objective was not to determine the PTA for patients with obesity but rather determine if there were any differences among weight quartiles. Moreover, our data may not be applicable in non-ICU patients who are underweight (weight: <40 kg) because our minimum weight was set as 40 kg, and pharmacokinetic data were derived from critically ill patients. Lastly, we have not further analyzed any toxicity profiles nor outcome data. These PTA tables will provide better guidance to clinicians who have different antimicrobial PD benchmarks (fT≥1×MIC vs. 100%fT≥1×MIC vs. fT≥4×MIC) for their critically ill patients undergoing CRRT.

## 4. Materials and Methods

This study was a post-hoc analysis of a previously published paper determining the influence of CRRT’s intensity (less intensive vs. intensive) on antibiotic exposure profiles [5]. Institutional review board approval was not required since pharmacokinetic and demographic data were applied to computer-generated “virtual” patients.

### 4.1. Pharmacokinetic Model and Simulations

The initial study [5] utilized one-compartment, first-order, and multiple-dose pharmacokinetic models to simulate antibiotic plasma concentration–time profiles based on demographic and CRRT dose information from the ATN trials [2,18]. Pertinent pharmacokinetic data in critically ill patients (Vd, unbound fraction, and nonrenal clearance (CL_NR_)) were collected from primary literature sources and incorporated in the MCS (Table 3). Beta-lactams (cefepime, ceftazidime, and piperacillin/tazobactam) and carbapenems (imipenem, ertapenem, and meropenem) were chosen for analysis because they were commonly used during the time of the ATN trial. The commonly recommended antibiotic dosing regimens for CRRT were simulated for 72 h in MCS. Drug concentration–time profiles were generated in a log-Gaussian distribution with preset limits using the mean and SD of the pharmacokinetic parameters outlined in Table 3 by the MCS (Crystal Ball, Oracle©, Santa Clara, CA, USA). The mean and SD of subject weight and delivered effluent rates from each study were used for that study’s MCS. Detailed descriptions of the PK model and MCS are included in the previous report [5].

### 4.2. Pharmacodynamic Targets

We used the Clinical and Laboratory Standards Institute (CLSI) susceptibility breakpoints against *P. aeruginosa* which are: 2 mg/L for meropenem and imipenem, 8 mg/L for cefepime and ceftazidime, and 16 mg/L for piperacillin (4 mg/L for tazobactam threshold). The susceptibility breakpoint for ertapenem against *S. pneumoniae* is 1 mg/L [35]. The PD targets were: ≥40%fT≥1×MIC of 2 mg/L for meropenem and imipenem (4×MIC = 8 mg/L), ≥40%fT≥1×MIC of 1 mg/L for ertapenem (4×MIC = 4 mg/L), ≥50%fT≥1×MIC of 16 mg/L for piperacillin (4×MIC = 64 mg/L), ≥50% fT>4 mg/L for tazobactam, and ≥60% fT≥1×MIC of 8 mg/L for cefepime and ceftazidime (4×MIC = 32 mg/L) over the first 72 h of antibiotic therapy [36,37]. Delattre and colleagues have recommended the use of %fT≥4×MIC as the benchmark for beta-lactams [36]. In order to implement in vitro, animal, and clinical data regarding optimal beta-lactam PD targets, we tested %fT≥MIC targets (1×MIC and 4×MIC) and 100%fT≥MIC in the present analysis.

### 4.3. Optimal Dosing Regimen

Drug dosing regimen was considered optimal if it reached a PTA of 90%, which is a standard threshold in simulation studies [5,23,30,38]. This means the virtual patients will achieve 90% of predetermined pharmacodynamic targets with simulated dosing regimens. Antibiotic toxicity profiles were not analyzed in this experiment, as the threshold for toxicity is poorly characterized [37,38].

### 4.4. Weight Quartile Analysis

The weight for 10,000 virtual subjects was limited to a minimum of 40 kg with no maximum limit set. The 10,000 virtual patients were organized by body weight, and their PTA analyses were divided into four quartiles. The lightest group was “Q1” (the 2500 virtual patients with the lowest weight) through the heaviest group called “Q4” (the 2500 virtual patients with the highest weight). Since there were 10,000 virtual subjects for each drug and dosing regimen and each was modeled separately, the weights within each quartile differ slightly between regimens.

## 5. Conclusions

Our post-hoc analysis shows that the patient’s weight influences antibiotic drugs’ pharmacodynamic target attainment related to antimicrobial efficacy. One-size-fits-all dosing should not be applied to large critically ill patients who might be obese, fluid overloaded, or both. This analysis does not include toxicity analysis but rather includes the PTA for 10,000 virtual patients to achieve different pharmacodynamic targets. Thus, we are not recommending any specific drug dosing regimen.

## Figures and Tables

**Table 1:** Probability of target attainment comparison among weight quartiles for key beta-lactams used in the ATN trial: Cefepime, ceftazidime, piperacillin, and tazobactam.

	ATN Less Intensive				ATN Intensive		
Weight Quartile	1×MIC	4×MIC	100% fT>1×MIC	Weight Quartile	1×MIC	4×MIC	100% fT>1×MIC
**Cefepime 1 g every 12 h**							
*Overall*	*100%*	*7.8%*	*10.8%*	*Overall*	***99.9%***	*2.3%*	*8.4%*
Q1 (40–70 kg)	100%	18.2%	31%	Q1 (40–70 kg)	99.8%	6.5%	24.4%
Q2 (70–82 kg)	100%	8.6%	9.6%	Q2 (70–82 kg)	100%	2.2%	7.7%
Q3 (82–95 kg)	100%	3.7%	2.3%	Q3 (82–96 kg)	100%	0.6%	1.3%
Q4 (95–177 kg)	100%	0.6%	0.4%	Q4 (96–204 kg)	100%	0.0%	0.1%
**Cefepime 1 g every 8 h**							
*Overall*	*100%*	*57.4%*	*15.5%*	*Overall*	*100%*	*33%*	*15.6%*
Q1 (40–70 kg)	100%	79.6%	43.7%	Q1 (40–70 kg)	100%	59.7%	44.9%
Q2 (70–82 kg)	100%	68.6%	14.2%	Q2 (70–82 kg)	100%	39.8%	13.5%
Q3 (82–95 kg)	100%	54.5%	4%	Q3 (82–96 kg)	100%	24.4%	3.8%
Q4 (95–189 kg)	100%	27.1%	0.2%	Q4 (96–213 kg)	100%	8.2%	0.4%
**Cefepime 2 g every 12 h**							
*Overall*	*100%*	*86.5%*	*56.3%*	*Overall*	*100%*	*77.2%*	*55.2%*
Q1 (40–70 kg)	100%	94.04%	90.3%	Q1 (40–70 kg)	100%	88.7%	88.1%
Q2 (70–82 kg)	100%	93.9%	71%	Q2 (70–82 kg)	100%	86.9%	70.6%
Q3 (82–95 kg)	100%	89.04%	46.4%	Q3 (82–96 kg)	100%	79.2%	44.9%
Q4 (95–180 kg)	100%	69.04%	17.3%	Q4 (96–183 kg)	100%	53.9%	17.0%
**Cefepime 2 g every 8 h**							
*Overall*	*100%*	*100%*	*57%*	*Overall*	*100%*	*99%*	*56.9%*
Q1 (40–70 kg)	100%	100%	92.4%	Q1 (40–70 kg)	100%	99.7%	92.4%
Q2 (70–82 kg)	100%	100%	72.6%	Q2 (70–82 kg)	100%	100.0%	71.4%
Q3 (82–96 kg)	100%	100%	45.6%	Q3 (82–96 kg)	100%	99.8%	46.2%
Q4 (96–185 kg)	100%	98.9%	17.3%	Q4 (96–217 kg)	100%	96.3%	17.5%
**Ceftazidime 1 g every 12 h**							
*Overall*	*100%*	*31.3%*	*31.2%*	*Overall*	*100%*	*16.9%*	*24.7%*
Q1 (40–70 kg)	100%	54.5%	51.8%	Q1 (40–70 kg)	100%	37.6%	46.3%
Q2 (70–82 kg)	100%	37%	34.8%	Q2 (70–82 kg)	100%	18.3%	29.3%
Q3 (82–95 kg)	100%	23.8%	25.7%	Q3 (82–96 kg)	100%	8.8%	16.5%
Q4 (95–200 kg)	100%	9.7%	12.5%	Q4 (96–204 kg)	99.9%	2.9%	6.8%
**Ceftazidime 2 g every 12 h**							
*Overall*	*100%*	*95.7%*	*81.1%*	*Overall*	*100%*	*88%*	*78.3%*
Q1 (40–70 kg)	100%	97.9%	93.2%	Q1 (40–70 kg)	100%	88.1%	78.5%
Q2 (70–82 kg)	100%	97.4%	86.7%	Q2 (70–82 kg)	100%	87.7%	78.4%
Q3 (82–95 kg)	100%	96.7%	81.0%	Q3 (82–96 kg)	100%	88%	78.2%
Q4 (95–183 kg)	100%	90.9%	63.5%	Q4 (96–193 kg)	100%	88.2%	78.3%
**Piperacillin 3 g every 12 h**							
*Overall*	*93.4%*	*30.7%*	*23.8%*	*Overall*	*91.9%*	*24.4%*	*20.5%*
Q1 (41–71 kg)	91%	38%	35.2%	Q1 (40–70 kg)	90.1%	33.0%	32.6%
Q2 (71–82 kg)	93.3%	33%	27.6%	Q2 (70–82 kg)	91.4%	25.8%	23.3%
Q3 (82–96 kg)	94.5%	28.9%	20.4%	Q3 (82–96 kg)	92.2%	22.9%	16.2%
Q4 (96–191 kg)	94.6%	22.9%	11.9%	Q4 (96–204 kg)	93.8%	16.1%	9.7%
**Piperacillin 4 g** **every 12 h**							
*Overall*	*96.3%*	*50%*	*42.8%*	*Overall*	*95.4%*	*44.6%*	*38.4%*
Q1 (40–70 kg)	94.7%	49%	55.7%	Q1 (40–70 kg)	93.8%	53.0%	51.2%
Q2 (70–82 kg)	95.3%	48.8%	46.8%	Q2 (70–82 kg)	94.8%	48.3%	43.6%
Q3 (82–95 kg)	97.3%	51.7%	40.8%	Q3 (82–96 kg)	96.2%	43.0%	34.6%
Q4 (95–184 kg)	97.8%	50.4%	28%	Q4 (96–213 kg)	96.6%	34.0%	24.0%
**Piperacillin 3 g** **every 8 h**							
*Overall*	*99%*	*61%*	*33.5%*	*Overall*	*98.8%*	*56.6%*	*33.1%*
Q1 (40–71 kg)	98.7%	66.1%	50.9%	Q1 (40–70 kg)	97.4%	62.0%	50.1%
Q2 (71–82 kg)	98.8%	63.5%	37.2%	Q2 (70–82 kg)	99.2%	60.2%	37.5%
Q3 (82–95 kg)	99%	59.8%	29%	Q3 (82–96 kg)	99.3%	56.6%	28.6%
Q4 (95–191 kg)	99.4%	54.4%	17%	Q4 (96–183 kg)	99.4%	47.3%	16.0%
**Piperacillin 4 g** **every 8 h**							
*Overall*	*99.5%*	*77.9%*	*54.6%*	*Overall*	*99.3%*	*75.1%*	*52.9%*
Q1 (40–70 kg)	99%	81%	72%	Q1 (40–70 kg)	98.5%	77.6%	69.4%
Q2 (70–82 kg)	99.5%	78.2%	59.2%	Q2 (70–82 kg)	99.3%	76.9%	59.2%
Q3 (82–96 kg)	99.5%	77.2%	49.2%	Q3 (82–96 kg)	99.6%	74.8%	49.0%
Q4 (96–206 kg)	99.8%	75.2%	37.9%	Q4 (96–217 kg)	99.7%	71.1%	33.7%
**Piperacillin 3 g** **every 6 h**							
*Overall*	*99.9%*	*80%*	*39.2%*	*Overall*	*99.8%*	*77.1%*	*37.9%*
Q1 (40–70 kg)	99.8%	83.6%	60%	Q1 (40–70 kg)	99.6%	80.4%	58.4%
Q2 (70–82 kg)	99.8%	81.6%	43.6%	Q2 (70–82 kg)	99.9%	77.9%	42.7%
Q3 (82–96 kg)	99.9%	79.4%	32.8%	Q3 (82–96 kg)	99.7%	78.4%	31.8%
Q4 (96–217 kg)	100%	75.5%	20.2%	Q4 (96–217 kg)	100%	71.5%	18.6%
**Piperacillin 4 g every 6 h**							
*Overall*	*99.9%*	*89.9%*	*60%*	*Overall*	*99.9%*	*88.5%*	*57.6%*
Q1 (40–71 kg)	99.8%	89.9%	77.9%	Q1 (40–70 kg)	99.8%	90.7%	76.2%
Q2 (71–82 kg)	100%	89.5%	66.2%	Q2 (70–82 kg)	99.8%	89.0%	63.4%
Q3 (82–95 kg)	99.9%	90.4%	56%	Q3 (82–96 kg)	99.9%	87.8%	54.5%
Q4 (95–184 kg)	100%	89.8%	39.8%	Q4 (96–217 kg)	100%	86.4%	36.0%
**Tazobactam 375 mg every 12 h**							
*Overall*	*76.8%*	*10%*	*3.6%*	*Overall*	*73%*	*5.4%*	*2.3%*
Q1 (40–71 kg)	79.4%	17.5%	7.2%	Q1 (40–70 kg)	76.5%	10.4%	4.6%
Q2 (71–82 kg)	78.2%	10.6%	3.6%	Q2 (70–82 kg)	76.1%	5.5%	2.4%
Q3 (82–95 kg)	76.4%	7.4%	2.3%	Q3 (82–96 kg)	72.9%	3.9%	1.6%
Q4 (95–199 kg)	73%	4.4%	1.2%	Q4 (96–202 kg)	66.2%	1.8%	0.7%
**Tazobactam 500 mg every 12 h**							
*Overall*	*84.7%*	*21.8%*	*8.5%*	*Overall*	*82.9%*	*14.8%*	*7%*
Q1 (40–71 kg)	85.4%	30.5%	13.8%	Q1 (40–70 kg)	84.5%	23.7%	13.1%
Q2 (71–82 kg)	84.2%	23.6%	9.6%	Q2 (70–82 kg)	84.5%	16.5%	7.7%
Q3 (82–96 kg)	85.7%	19.6%	6.6%	Q3 (82–96 kg)	81.4%	12.5%	5.0%
Q4 (96–187 kg)	83.6%	13.4%	4.1%	Q4 (96–204 kg)	80.6%	6.7%	2.2%
**Tazobactam 375 mg every 8 h**							
*Overall*	*89.1%*	*27.9%*	*4.7%*	*Overall*	*87.8%*	*20.9%*	*4.6%*
Q1 (40–71 kg)	89.5%	36.1%	8.8%	Q1 (40–70 kg)	88.6%	30.3%	9.2%
Q2 (71–82 kg)	89.4%	31.8%	4.9%	Q2 (70–82 kg)	87.6%	25.0%	5.5%
Q3 (82–95 kg)	88.9%	25.1%	3.2%	Q3 (82–96 kg)	88.1%	17.2%	2.4%
Q4 (95–222 kg)	88.5%	18.7%	1.9%	Q4 (96–184 kg)	86.6%	11.2%	1.3%
**Tazobactam 375 mg every 6 h**							
*Overall*	*93.4%*	*44.8%*	*6.6%*	*Overall*	*93.6%*	*38.8%*	*6.2%*
Q1 (40–70 kg)	93.8%	53.8%	11.5%	Q1 (40–70 kg)	94.2%	50.5%	12.0%
Q2 (70–82 kg)	92.8%	47.5%	6.7%	Q2 (70–82 kg)	93.6%	42.1%	6.8%
Q3 (82–95 kg)	94%	43%	5.2%	Q3 (82–96 kg)	94.1%	37.1%	4.0%
Q4 (95–225 kg)	93%	35.1%	2.9%	Q4 (96–185 kg)	92.4%	25.3%	1.9%
**Tazobactam 500 mg every 8 h**							
*Overall*	*93.2%*	*45.5%*	*11.7%*	*Overall*	*92.3%*	*38%*	*10.3%*
Q1 (40–70 kg)	93%	56.2%	19.3%	Q1 (40–70 kg)	92.5%	48.5%	17.8%
Q2 (70–82 kg)	93.7%	48.6%	13.6%	Q2 (70–82 kg)	92.9%	42.6%	10.5%
Q3 (82–96 kg)	92.3%	43.2%	8.8%	Q3 (82–96 kg)	92.1%	35.5%	8.4%
Q4 (96–181 kg)	93.4%	34.2%	5%	Q4 (96–181 kg)	91.6%	25.5%	4.6%
**Tazobactam 500 mg every 6 h**							
*Overall*	*96.1%*	*61.3%*	*13.3%*	*Overall*	*95.8%*	*55.3%*	*12.3%*
Q1 (40–71 kg)	96%	68.9%	22.6%	Q1 (40–70 kg)	95.8%	64.8%	20.1%
Q2 (71–82 kg)	96%	63.8%	14.8%	Q2 (70–82 kg)	96.2%	60.5%	13.5%
Q3 (82–96 kg)	96.2%	59.4%	9.6%	Q3 (82–96 kg)	96.0%	53.3%	10.1%
Q4 (96–182 kg)	96.4%	53%	6.4%	Q4 (96–209 kg)	94.9%	42.6%	5.6%

Shaded to represent probability of target attainment ≥90% (green), 60 < 90% (orange), and <60% (red).

**Table 2:** Probability of target attainment comparison among weight quartiles for key carbapenems used in the ATN trial: ertapenem, imipenem, and meropenem.

	ATN Less Intensive				ATN Intensive		
Wt. Quartile	1×MIC	4×MIC	100% fT≥1×MIC	Wt. Quartile	1×MIC	4×MIC	100% fT≥1×MIC
**Ertapenem 1 g every 24 h (MIC 1)**							
*Overall*	*100%*	*100%*	*99.72%*	*Overall*	*100%*	*99.98%*	*99.17%*
Q1 (40–70 kg)	100%	100%	99%	Q1 (40–70 kg)	100%	100%	97.5%
Q2 (70–82 kg)	100%	100%	99.9%	Q2 (70–82 kg)	100%	100%	99.6%
Q3 (82–96 kg)	100%	100%	100%	Q3 (82–96 kg)	100%	100%	99.7%
Q4 (96–204 kg)	99.9%	99.9%	100%	Q4 (96–212 kg)	99.9%	99.8%	99.8%
**Ertapenem 1 g every 24 h (MIC 2)**							
*Overall*	*100%*	*98.2%*	*93.7%*	*Overall*	*98.2%*	*87.32%*	*87.73%*
Q1 (40–70 kg)	100%	99.7%	91.2%	Q1 (40–70 kg)	100%	98.6%	82%
Q2 (70–82 kg)	100%	99.6%	97.6%	Q2 (70–82 kg)	100%	96%	91.8%
Q3 (82–96 kg)	100%	98.8%	97.8%	Q3 (82–96 kg)	100%	89.8%	93.4%
Q4 (96–213 kg)	99.9%	94.6%	87.2%	Q4 (96–212 kg)	99.9%	64.7%	83.7%
**Imipenem 500 mg every 12 h**							
*Overall*	*98%*	*3.3%*	*5.8%*	*Overall*	*97.3%*	*1.8%*	*3.6%*
Q1 (40–70 kg)	95%	3.8%	3.2%	Q1 (40–70 kg)	92.2%	2.7%	2.2%
Q2 (70–82 kg)	98%	3.8%	5.5%	Q2 (70–82 kg)	98.1%	2.0%	3.9%
Q3 (82–95 kg)	99.2%	3.5%	7.9%	Q3 (82–96 kg)	99.2%	1.4%	3.9%
Q4 (95–199 kg)	99.9%	2.1%	6.4%	Q4 (96–201 kg)	99.6%	1.1%	4.4%
**Imipenem 500 mg every 8 h**							
*Overall*	*100%*	*40%*	*39.9%*	*Overall*	*100%*	*32.8%*	*33.4%*
Q1 (40–71 kg)	100%	44%	36.2%	Q1 (40–70 kg)	100%	36.2%	29.2%
Q2 (71–82 kg)	100%	43.7%	46.8%	Q2 (70–82 kg)	100%	36.2%	38.2%
Q3 (82–95 kg)	100%	39.2%	46.2%	Q3 (82–96 kg)	100%	33.9%	38.9%
Q4 (95–196 kg)	100%	33.2%	30.4%	Q4 (96–212 kg)	100%	24.6%	27.2%
**Imipenem 500 mg every 6 h**							
*Overall*	*100%*	*78.3%*	*61.6%*	*Overall*	*97.3%*	*74.6%*	*60%*
Q1 (40–71 kg)	100%	80.5%	71.3%	Q1 (40–70 kg)	100%	77.5%	68.1%
Q2 (71–82 kg)	100%	80%	71.7%	Q2 (70–82 kg)	100%	77.4%	70.6%
Q3 (82–95 kg)	100%	78%	61.8%	Q3 (82–96 kg)	100%	75%	60.7%
Q4 (95–191 kg)	100%	74.8%	41.8%	Q4 (96–187 kg)	100%	68.5%	40.9%
**Imipenem 1 g every 8 h**							
*Overall*	*100%*	*98%*	*87%*	*Overall*	*100%*	*97.3%*	*82.3%*
Q1 (40–71 kg)	100%	96.8%	71.4%	Q1 (40–70 kg)	100%	96.6%	65.6%
Q2 (71–82 kg)	100%	98.4%	87%	Q2 (70–82 kg)	100%	96.8%	81.0%
Q3 (82–96 kg)	100%	98.8%	93.5%	Q3 (82–96 kg)	100%	98.1%	88.9%
Q4 (96–193 kg)	100%	98.2%	96%	Q4 (96–202 kg)	100%	97.8%	93.8%
**Meropenem 500 mg every 12 h**							
*Overall*	*97.6%*	*63.3%*	*45.7%*	*Overall*	*97.4%*	*58.1%*	*45.7%*
Q1 (40–71 kg)	96.1%	66.4%	58.4%	Q1 (40–70 kg)	95.8%	65.1%	54.6%
Q2 (71–82 kg)	97.6%	65.8%	52.4%	Q2 (70–82 kg)	96.8%	60.0%	47.9%
Q3 (82–96 kg)	97.9%	63.8%	43.4%	Q3 (82–96 kg)	98.1%	57.8%	40.9%
Q4 (96–173 kg)	98.8%	57.2%	28.6%	Q4 (96–217 kg)	98.9%	49.6%	24.5%
**Meropenem 500 mg every 8 h**							
*Overall*	*99.8%*	*84.8%*	*57.9%*	*Overall*	*99.7%*	*82.6%*	*55.8%*
Q1 (40–71 kg)	99.5%	87.2%	77.6%	Q1 (40–70 kg)	99.2%	85.6%	74.1%
Q2 (71–82 kg)	99.8%	85.08%	64.9%	Q2 (70–82 kg)	99.8%	83.6%	63.8%
Q3 (82–96 kg)	100%	84.9%	55.1%	Q3 (82–96 kg)	99.7%	82.2%	52.8%
Q4 (96–189 kg)	99.8%	81.8%	33.9%	Q4 (96–206 kg)	99.9%	78.8%	32.5%
**Meropenem 1 g every 12 h**							
*Overall*	*99.4%*	*90.6%*	*82%*	*Overall*	*99.2%*	*89.8%*	*79.5%*
Q1 (40–70 kg)	98.6%	88.6%	77.6%	Q1 (40–70 kg)	98%	87.5%	74.2%
Q2 (70–82 kg)	99.3%	90.7%	84.4%	Q2 (70–82 kg)	99%	90%	81%
Q3 (82–95 kg)	99.8%	91.8%	85.8%	Q3 (82–96 kg)	100%	90.6%	82.8%
Q4 (95–183 kg)	99.9%	91.1%	80%	Q4 (96–206 kg)	100%	90.8%	79.8%
**Meropenem 1 g every 8 h**							
*Overall*	*100%*	*98.1%*	*92.2%*	*Overall*	*99.9%*	*97.6%*	*90.8%*
Q1 (40–70 kg)	99.9%	97.3%	91.5%	Q1 (40–70 kg)	99.8%	97%	90.3%
Q2 (70–82 kg)	100%	98%	94.2%	Q2 (70–82 kg)	100%	100%	93.8%
Q3 (82–95 kg)	100%	98.9%	94.8%	Q3 (82–96 kg)	100%	97.9%	93%
Q4 (95–195 kg)	100%	98.2%	88.3%	Q4 (96–202 kg)	100%	97.9%	86.2%
**Meropenem 2 g every 12 h**							
*Overall*	*99.8%*	*98.1%*	*91.4%*	*Overall*	*99.7%*	*97.4%*	*89.5%*
Q1 (40–71 kg)	99.6%	97.1%	86.5%	Q1 (40–70 kg)	99.4%	95.8%	83.2%
Q2 (71–82 kg)	99.8%	97.9%	89.7%	Q2 (70–82 kg)	99.6%	97.5%	89.4%
Q3 (82–95 kg)	99.8%	98.3%	93.3%	Q3 (82–96 kg)	99.8%	97.8%	91.6%
Q4 (95–199 kg)	100%	99%	96%	Q4 (96–206 kg)	100%	98.6%	93.7%

Shaded to represent probability of target attainment: ≥90% (green), 60 ≤ 89% (orange), and <60% (red).

**Table 3:** Adapted pharmacokinetic parameters used in Monte Carlo simulations [5].

Drug [Ref]	Cefepime [18,19,20,21,22,23]	Ceftazidime [24,25,26,27,28,29]	Ertapenem [5,30]	Imipenem [5,30]	Meropenem [5,30]	Piperacillin [23,31,32,33,34]	Tazobactam [23,33]
Vd (L/kg)	0.48 ± 0.24 (0.16–1.11)	0.34 ± 0.20 (0.13–1.1)	0.19 ± 0.07 (0.13–0.34)	0.34 ± 0.1 (0.21−0.63)	0.41 ± 0.18 (0.08−1.07)	0.40 ± 0.21 (0–1.11)	0.50 ± 0.37 (0–2.13)
Free Fraction	0.79 ± 0.09 (0.72–0.85)	0.86 ± 0.05 (0.75–0.94)	0.25 ± 0.45 (0−1)	0.8 ± 0.16 (0−1)	0.79 ± 0.09 (0−1)	0.76 ± 0.2 (0–1)	0.74 ± 0.27 (0–1)
NR CL (mL/min)	24.33 ± 11.25 (13–44)	15.9 ± 9.9 (8–37.7)	11 ± 3 (10−19)	100.5 ± 28 (53−160)	54.9 ± 49 (0−251)	48.5 ± 37 (0–187)	40.4 ± 70 (0–381)
Sieving coefficient	0.67 ± 0.13 (0–1)	0.85 ± 0.05 (0–1)	0.2 ± 0.06 (0−1)	0.57 ± 0.1 (0−1)	0.63 ± 0.13 (0−1)	0.6 ± 0.28 (0–1)	0.8 ± 0.36 (0–1)
r^2^ weight and Vd	0.4197	0.0237	0.3318	0.17	0.1435	0.0567	0.0049
r^2^ weight and NR CL	0.038	0.1254	0.1156	0.013	0.0072	0.036	0.0098
Weight ± SD (kg)	Less intensive: 84.1 ± 18.9; Intensive: 84.1 ± 19.6						
CRRT % delivered	Less intensive: 0.95 ± 0.35 (0–1); Intensive: 0.89 ± 0.39 (0–1)						
Q_eff_ (mL/kg/h)	Less intensive: 22 ± 6.1 (0–47.5) vs. Intensive: 35.8 ± 6.4 (0–47.5)						
Q_rep_ (L/h)	Less intensive: 0.83 ± 0.25 (0.33–1.33); Intensive: 0.89 ± 0.39 (0–1)						

All values are mean ± standard deviation (minimum–maximum limits). Abbreviations: CL = clearance; NR = nonrenal; r^2^ = correlation; Vd = volume of distribution; Q_eff_ = effluent flow rate; Q_rep_ = replacement fluid rate.

## Data Availability

Data are available and stored at the University of Michigan College of Pharmacy.
